# An orally administrable hydrogen-bonded organic framework (HOF)-based nanoreactor to reprogram metabolism for senescence intervention

**DOI:** 10.1093/nsr/nwag074

**Published:** 2026-02-03

**Authors:** Haochen Zhang, Junlin Ya, Jinsong Ren, Xiaogang Qu

**Affiliations:** Laboratory of Chemical Biology and State Key Laboratory of Rare Earth Resource Utilization, Changchun Institute of Applied Chemistry, Chinese Academy of Sciences, Changchun 130022, China; School of Applied Chemistry and Engineering, University of Science and Technology of China, Hefei 230026, China; Laboratory of Chemical Biology and State Key Laboratory of Rare Earth Resource Utilization, Changchun Institute of Applied Chemistry, Chinese Academy of Sciences, Changchun 130022, China; School of Applied Chemistry and Engineering, University of Science and Technology of China, Hefei 230026, China; Laboratory of Chemical Biology and State Key Laboratory of Rare Earth Resource Utilization, Changchun Institute of Applied Chemistry, Chinese Academy of Sciences, Changchun 130022, China; School of Applied Chemistry and Engineering, University of Science and Technology of China, Hefei 230026, China; Laboratory of Chemical Biology and State Key Laboratory of Rare Earth Resource Utilization, Changchun Institute of Applied Chemistry, Chinese Academy of Sciences, Changchun 130022, China; School of Applied Chemistry and Engineering, University of Science and Technology of China, Hefei 230026, China

**Keywords:** hydrogen-bonded organic frameworks, senescence, oral catalytic therapy, gut metabolome

## Abstract

Aging poses significant health challenges due to functional decline and increased disease susceptibility. Modulating age-related metabolic dysregulation offers a promising therapeutic avenue. However, the efficacy of oral therapies is severely hampered by the degradation of active agents in the harsh gastrointestinal (GI) tract. Here, we report an orally administrable hydrogen-bonded organic framework (HOF)-based nanoreactor (MAO@NADH@PB@O-HOF@PEG) designed to intervene in senescence by modulating metabolic pathways. The HOF scaffold exhibits exceptional stability in ultra-acidic conditions and boiling water, providing a robust shield for its encapsulated delicate cargo against GI degradation. This protection enables prolonged *in situ* catalysis within the intestine, leading to reprogramming of the gut metabolism by a catalytic degradation of the aging-elevated metabolite isoamylamine (IAA) in the intestine, serum and cerebrospinal fluid (CSF). The apoptosis and inflammation driven by elevated IAA levels are mitigated through the catalytic degradation of IAA by the HOF-based anti-aging nanoreactor. Oral administration of MAO@NADH@PB@O-HOF@PEG to aged mice significantly alleviated cognitive decline, neuronal damage and neuroinflammation. This study highlights the potential of robust HOF materials in the development of advanced oral delivery systems and geroprotective therapeutics.

## Teaser

A robust, orally administrable hydrogen-bonded organic framework nanoreactor shields enzymes from gastrointestinal degradation, enabling gut metabolic reprogramming to alleviate age-related cognitive decline and neuroinflammation.

## INTRODUCTION

Aging is an inevitable multifactorial process that leads to age-related cognitive impairments, loss of function, increased disease susceptibility and death, creating a need for interventions that delay aging to greatly benefit health [1–3]. Despite decades of research, effectively altering this process remains challenging. Strategies focused on identifying geroprotective compounds that target specific pathways have shown low efficiency, and the use of dietary additives may induce undesirable side effects such as potential tumorigenesis [4,5]. Emerging evidence indicates that numerous metabolites, including those derived from gut microbiomes [such as δ-valerobetaine, phenylacetylglutamine or isoamylamine (IAA)], regulate host longevity, gene expression and signaling pathways, playing key physiological and pathophysiological roles [6]. Therefore, modulating the *in vivo* levels of these metabolites to impede senescence-driving processes, such as metabolite-induced senescence, holds significant therapeutic potential [7–9]. However, broad interventions such as the use of broad-spectrum antibiotics or fecal microbiota transplantation involve the reshaping of entire microbial ecosystems, raising concerns about donor dependency, safety (e.g. pathogen co-transplantation risk), long-term dysbiosis and antibiotic resistance [6,10]. Therefore, developing novel strategies for precise, localized modulation of metabolic pathways within the gastrointestinal (GI) tract is highly desirable.

Oral catalytic therapy has emerged as a promising approach for such targeted interventions, given its patient-friendly advantages [11–13]. Indeed, recent advances have validated this concept, utilizing oral nanozymes and nanocarriers to modulate gut inflammation or metabolic pathways [14–16]. Oral administration is widely regarded as the safest, most convenient and most cost-effective delivery route, eliminating risks associated with injections and enhancing patient compliance, particularly for chronic conditions [17–20]. However, a wide range of therapeutic agents—including drugs, enzymes, transition metal catalysts and functionalized antibodies—can be degraded by extreme pH and digestive enzymes, leading to low bioavailability, and necessitating higher dosages that can exacerbate the gastrointestinal burden [21–24]. This challenge underscores the urgent need for robust delivery platforms constructed from materials that are cost-effective, tunable, biodegradable, biocompatible and readily scalable to support the expanding pipeline of oral catalytic therapies.

Recently, hydrogen-bonded organic frameworks (HOFs) have emerged as ideal candidates to address these challenges for biocatalysis and drug delivery [25,26]. The mild synthesis conditions enable the encapsulation of delicate biomolecules, such as enzymes, while preserving their native structures and overcoming common limitations of poor stability and storage [27–30]. Certain HOFs exhibit exceptional thermal stability and retain structural integrity under humid or extreme acidic environments [31,32]. This inherent robustness allows the framework to effectively protect encapsulated payloads from the harsh *in vivo* conditions of the GI tract, addressing the key oral delivery barriers [27,33]. Upon encapsulation, the HOF composites not only exhibit markedly enhanced stability compared to the free cargo but also, critically, retain biocatalytic performance [27,34]. Moreover, compared to other framework materials, such as metal-organic frameworks (MOFs) that degrade rapidly in biological milieu, or covalent organic frameworks (COFs) with limited loading capacity, HOFs uniquely combine outstanding stability, high biocompatibility and significant cargo-loading capacity [35,36]. Furthermore, the metal-free nature of HOFs enhances their biosafety by circumventing the risk of metal leaching [35]. Thus, HOFs represent a truly promising new platform for orally administered therapies, expanding the materials toolbox beyond conventional polymers, hydrogels and liposomes [11].

**Scheme 1.**
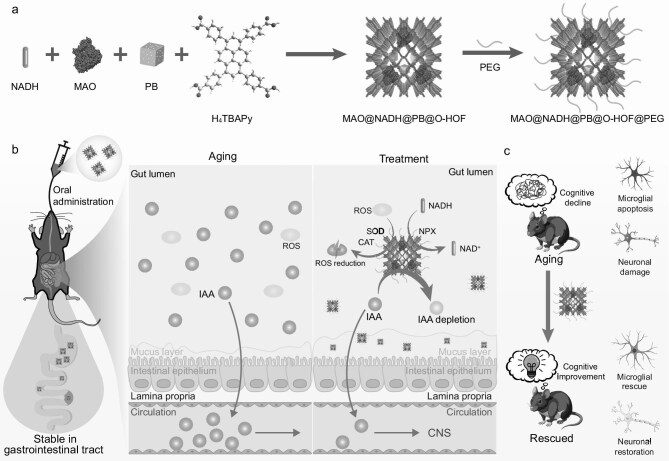
(a) Schematic illustration of the construction of the oral nanoreactor MAO@NADH@PB@O-HOF@PEG. (b) Therapeutic mechanism and (c) overall therapeutic outcomes of MAO@NADH@PB@O-HOF@PEG. Parts of this figure were adapted from SciDraw (DOIs: 10.5281/zenodo.4717443 and 10.5281/zenodo.5348394).

Herein, we report an orally administrable HOF-based anti-aging nanoreactor (designated MAO@NADH@PB@O-HOF@PEG), engineered to modulate metabolic dysregulation for the intervention of senescence (Scheme 1). The nanoreactor is constructed from a biocompatible oral HOF (O-HOF) with exceptional acid stability and thermal stability, which serves as a robust shield for the cargo. The O-HOF scaffold effectively protects encapsulated enzymatic cargo (monoamine oxidase, MAO), nanozymes (Prussian blue, PB) and bioactive molecules (nicotinamide adenine dinucleotide, NADH) from degradation in simulated gastric fluid (SGF) and simulated intestinal fluid (SIF). As a clinical oral antidote approved by the US Food and Drug Administration (FDA) [37], PB with multienzyme-like activity including superoxide dismutase (SOD), catalase (CAT) and NADH peroxidase (NPX) ameliorated oxidative stress prevalent in the aging gut [38], which in turn mitigates gut barrier permeability and limits the systemic circulation of IAA [39]. NADH serves as both a substrate for NPX and an intrinsic anti-aging molecule [40,41]. Furthermore, surface functionalization with polyethylene glycol (PEG), a prominent FDA-approved polymer [42], provided extended intestinal residence time for efficient *in situ* catalysis [43,44]. Following oral administration to aged mice, the HOF-based catalytic nanoreactor effectively decreased IAA levels in the intestine, serum and cerebrospinal fluid (CSF). The age-related cognitive decline and neuronal damage of mice were alleviated, and both apoptosis and neuroinflammation were mitigated. This study provides valuable perspectives for developing HOFs as a robust and versatile platform for oral therapies targeting age-associated pathologies.

## RESULTS

**Figure 1.**
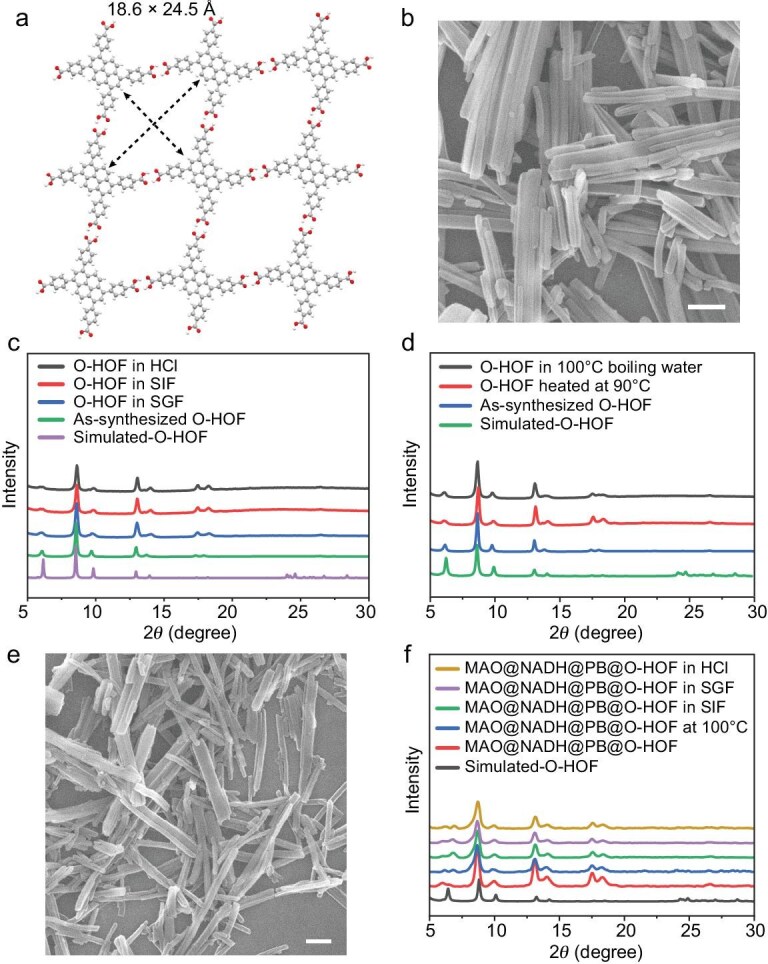
(a) 1D channels of the crystal structure of O-HOF. (b) SEM image of O-HOF. Scale bar = 300 nm. (c) PXRD patterns of as-synthesized O-HOF and samples treated with different solutions. (d) PXRD patterns of O-HOF heated at different temperatures. (e) SEM image of MAO@NADH@PB@O-HOF. Scale bar = 300 nm. (f) PXRD patterns of MAO@NADH@PB@O-HOF after different treatments.

The ligand 1,3,6,8-tetrakis (p-benzoic acid) pyrene (H_4_TBAPy), a planar molecule featuring a very large π-conjugated system and four carboxylic acid groups, was selected as the building block to construct highly crystalline HOFs (denoted as PFC-1 or O-HOF). O-HOF possesses channel-like mesopores with dimensions of 18.6 × 24.5 Å (Fig. 1a). The neighboring layers of O-HOF interact with adjacent layers through face-to-face π–π stacking interactions (Fig. S1). The scanning electron microscopy (SEM) images showed that O-HOF possesses a needle-shaped morphology (Fig. 1b). Powder X-ray diffraction (PXRD) analysis revealed that the experimental pattern of O-HOF matches the simulated pattern of O-HOF, demonstrating its successful synthesis and high phase purity (Fig. 1c). The accessible porosity of O-HOF was confirmed by the guest inclusion method as described recently [45,46] (Fig. S2). As previously reported, the π–π interaction energies of the O-HOF dimer and trimer were calculated as −20.351 and −40.607 kcal mol^−1^, respectively [31]. These values are much greater than the typical hydrogen bonding energy of −18.986 kcal mol^−1^, demonstrating the vital role of π–π stacking in contributing to its structural stability (Fig. S1). Thus, O-HOF exhibits high chemical stability as confirmed by its maintained PXRD patterns and crystallinity after exposure to concentrated hydrochloric acid (12 M HCl), SGF and SIF solutions (Fig. 1c). Furthermore, the PXRD patterns of O-HOF exhibited no significant changes after treatment with hot water at 90°C and 100°C, underscoring its robust thermal stability and potential suitability for oral administration (Fig. 1d). To verify the stability of O-HOF in aqueous environments, we examined the O-HOF by SEM after incubation in deionized water for 72 h. As shown in Fig. S3, the SEM images revealed that the needle-like morphology and structural integrity of O-HOF were well preserved, demonstrating its structural stability in aqueous environments.

The construction of MAO@NADH@PB@O-HOF was initiated by preparing the PB nanoparticles [47]. The SEM images revealed that PB nanoparticles exhibited a well-defined square morphology and high monodispersity (Figs S4a and S5), with an average diameter of 54.8 ± 8.9 nm (Fig. S4b). Next, MAO@NADH@PB@O-HOF was synthesized using a previously reported method [48]. The successful formation and structural integrity of MAO@NADH@PB@O-HOF were confirmed by SEM and PXRD analysis. The SEM image (Fig. 1e) of MAO@NADH@PB@O-HOF revealed a nanosized, cuboid-like morphology comparable to that of the parent O-HOF. Furthermore, the PXRD patterns of MAO@NADH@PB@O-HOF were similar to those of O-HOF, indicating that the architecture and crystallinity of O-HOF were maintained throughout the encapsulation process (Fig. 1f). We further verified the cargo incorporation by energy-dispersive X-ray spectroscopy (EDS) mapping (Fig. S6). The elemental maps revealed that the Fe signals (from PB) were detectable throughout the framework architecture. The spatial co-localization of Fe with C, N and O provides additional support for the successful *in situ* encapsulation of PB within the O-HOF matrix. The decrease of inclusion efficiency in the guest inclusion assay of MAO@NADH@PB@O-HOF also suggested the successful encapsulation of cargo (Fig. S7). To evaluate its stability under physiological conditions relevant to oral administration, MAO@NADH@PB@O-HOF was incubated in SGF and SIF. The PXRD patterns of the treated MAO@NADH@PB@O-HOF (Fig. 1f) showed no significant changes, confirming the structural stability of O-HOF and its function in protecting the encapsulated components against degradation by GI fluids. The PXRD patterns of MAO@NADH@PB@O-HOF were retained after treatment in water at 100°C, highlighting its notable thermal stability (Fig. 1f). To validate the structural stability of MAO@NADH@PB@O-HOF, we performed SEM characterization after incubation in water for 0 and 72 h. As shown in Fig. S8, the nanoreactors retained their original morphology and structural integrity, confirming their robustness in aqueous environments. Collectively, these results indicate the successful construction of the MAO@NADH@PB@O-HOF nanoreactors and their structural stability in aqueous environments.

Although the O-HOF scaffold provides robust protection, a major challenge for oral therapeutics is poor oral bioavailability in the GI tract, primarily due to the limited intestinal retention and harsh GI environment. PEG has been widely used to promote mucoadhesion via interpenetrating network effects, thereby enhancing intestinal retention [43,44]. Moreover, PEG-decorated nanoparticles evade cells by stealth and are minimally internalized, which enhances the biosafety of nanoparticles for intestinal catalysis by minimizing systemic absorption [49,50]. Notably, PEG is generally regarded as safe by the FDA and is the prominent FDA-approved polymer for bioconjugation [42]. Therefore, considering these advantages, PEG was selected to modify the MAO@NADH@PB@O-HOF to improve its intestinal retention. MAO@NADH@PB@O-HOF@PEG was synthesized according to previously reported methods [43,51], and its successful formation was verified by Fourier-transform infrared (FTIR) spectroscopy (Fig. S9). Subsequently, the loading contents of MAO@NADH@PB@O-HOF@PEG were quantified using methods tailored to each component. Specifically, upon dismantling the HOF framework, the encapsulated MAO was quantified as 115.4 μg/mg via sodium dodecyl sulfate-polyacrylamide gel electrophoresis (SDS-PAGE) (Fig. S10). The NADH loading (48.3 μg/mg) was calculated based on the residual concentration in the supernatant using an NAD^+^/NADH kit. Finally, the PB content (18.6 μg/mg) was determined by measuring the Fe concentration via inductively coupled plasma mass spectrometry (ICP-MS) after digesting the composite in aqua regia. To evaluate the protective capacity of the O-HOF, the MAO@NADH@PB@O-HOF@PEG was sequentially exposed to SGF and SIF. Subsequent SDS-PAGE analysis, performed upon HOF disassembly, revealed that the MAO remained intact (Fig. S10), indicating the robust protective capability of the O-HOF framework. Taken together, these results underscore the promising potential of MAO@NADH@PB@O-HOF@PEG for application within the *in vivo* GI environment.

**Figure 2.**
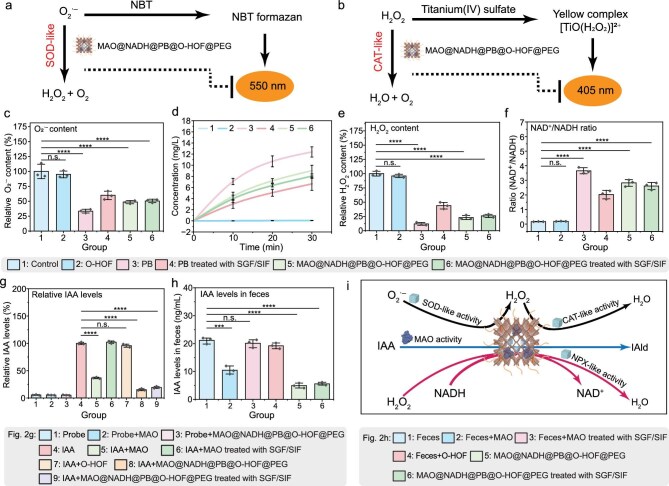
(a) Schematic illustration of SOD-like activity detection and (b) CAT-like activity detection. (c) Scavenging ability of different formulations against O_2_^·–^. (d) Time-dependent oxygen generation curves from H_2_O_2_ decomposition. (e) Scavenging capability against H_2_O_2_. (f) NPX-like activity assessment by monitoring the NAD^+^/NADH ratio. Phosphate buffered saline (PBS) buffer was used as the control. (g) *In vitro* catalytic degradation efficiency of IAA with free MAO, empty O-HOF and nanoreactor controls before and after SGF/SIF treatments. (h) Catalytic degradation of IAA in mouse feces. (i) Schematic mechanism of the multi-enzyme cascade system constructed by MAO@NADH@PB@O-HOF@PEG. The encapsulated MAO is responsible for the catalytic degradation of IAA. Concurrently, the PB component functions as a ROS scavenger: its SOD-like activity dismutates O_2_^·–^ into H_2_O_2_, which is subsequently eliminated by CAT-like activity and NPX-like activity (consuming loaded NADH to produce NAD^+^ and H_2_O). Data represent mean ± SD (*n* = 3). (n.s., not significant, *P < 0.05, **P < 0.01, ***P < 0.001, ****P < 0.0001).

As previously reported, oxidative stress in the gut tends to increase during aging, which can have detrimental effects on barrier integrity, gut microbiota and immune function [38]. In recent years, PB nanomaterials have exhibited neuroprotection and significant therapeutic effects for treating enteritis [37,52,53]. As an FDA-approved antidote with excellent biosafety [37], PB exhibits intrinsic enzyme-mimicking catalytic functions, including SOD-like, CAT-like and NPX-like activities [52,53]. Subsequently, we evaluated the multi-enzyme mimetic performance of nanoreactor MAO@NADH@PB@O-HOF@PEG compared to free PB nanoparticles and empty HOF controls (Fig. 2). We employed an SOD assay kit to assess the SOD-like activity of the nanoreactor (Fig. 2a). The nitrotetrazolium blue chloride (NBT) interacts with superoxide anion radical (O_2_^·–^) to form NBT formazan, which exhibits strong light absorption at 550 nm. The introduction of materials with SOD-like activity effectively reduces the concentration of substrate O_2_^·–^, consequently diminishing the formation of NBT formazan and leading to a decrease in absorbance intensity at 550 nm. The results revealed that the O-HOF scaffold itself possessed minimal SOD-like activity (Fig. 2c). While bare PB showed significant scavenging effects, its activity decreased remarkably after sequential treatment, first in SGF and then in SIF. Notably, the nanoreactor exhibited robust tolerance to SGF/SIF, preserving its reactive oxygen species (ROS)-scavenging performance. This suggests that the encapsulation can effectively protect the enzymatic core from environmental degradation. Subsequently, the CAT-like activity was evaluated by two methods: monitoring the generation of oxygen (O_2_) from hydrogen peroxide (H_2_O_2_) decomposition (Fig. 2d) and a colorimetric assay using titanium sulfate (Fig. 2b and e). Although bare PB displayed a high initial reaction rate, its activity declined significantly after exposure to SGF and SIF (Fig. 2d). Conversely, the MAO@NADH@PB@O-HOF@PEG nanoreactor retained stable catalytic performance under identical conditions, demonstrating that the O-HOF shell serves as a protective barrier to maintain the bioactivity of the inner enzymatic core. The protective effect was further verified by the titanium sulfate assay (Fig. 2b), which measures H_2_O_2_ consumption. As essential controls, the results demonstrated that the HOF alone exhibited no detectable catalytic activity (Fig. 2e). While the catalytic performance of bare PB was compromised by the SGF/SIF treatment, the MAO@NADH@PB@O-HOF@PEG nanoreactor retained its potent H_2_O_2_-scavenging activity. This finding offers additional evidence that encapsulation can protect enzymatic activity within the gastrointestinal tract. Furthermore, the NPX-like activity of the MAO@NADH@PB@O-HOF@PEG nanoreactor was validated by directly monitoring the generation of the product NAD⁺ using an NAD⁺/NADH assay kit, as evidenced by an increased NAD⁺/NADH ratio (Fig. 2f). As shown in Fig. 2f, the O-HOF shell was inactive. While the activity of bare PB decreased after SGF/SIF incubation, the nanoreactor maintained stable performance, confirming the shell’s protective role. These findings highlight the multi-enzyme mimetic activity of the MAO@NADH@PB@O-HOF@PEG nanoreactor, as well as its retained enzymatic activity, even in a simulated harsh GI environment.

Previous research has demonstrated that the metabolite IAA is enriched in the GI tract of aged mice and elderly individuals, and its elevated levels cause age-related cognitive decline [54]. To address this, we investigated the ability of the nanoreactor to catalytically degrade IAA (Fig. 2g and Fig. S11). As shown in Fig. 2g, while free MAO effectively catalyzed IAA decomposition initially, its activity was completely abolished following sequential SGF/SIF treatment. In contrast, the MAO@NADH@PB@O-HOF@PEG nanoreactor exhibited potent catalytic activity regardless of SGF/SIF treatment, indicating sustained enzymatic activity. Concurrently, we observed the generation of the catalytic product, the corresponding aldehyde, isoamyl aldehyde (IAld) (Fig. S12), indicating that the nanoreactor effectively catalyzed the reaction of IAA degradation. Additionally, as shown in Fig. S13, compared with free MAO at an equivalent protein concentration (quantified by SDS-PAGE), the encapsulated MAO in the nanoreactor (both before and after PEGylation) demonstrated higher IAA degradation efficiency. This activity enhancement is likely due to the confinement effect of the HOF framework—which stabilizes the active conformation and prevents aggregation—and the effective alleviation of product inhibition [34,55–57]. These results indicate that the framework effectively protects the enzyme, confirming that both the encapsulation and PEGylation processes preserve its bioactivity and highlighting the mild nature of our synthetic method. Furthermore, the nanoreactor also displayed high functional stability, retaining the majority of its catalytic activity for IAA degradation after 3 days of storage in deionized water (Fig. S13). This robust catalytic activity was further confirmed in a more biologically relevant milieu using fecal supernatant from aged mice. While the free MAO treated with SGF/SIF failed to degrade IAA due to inactivation (Fig. 2h), the nanoreactor could still effectively reduce IAA levels under the same SGF/SIF-treatment (Fig. 2h). Furthermore, the empty O-HOF showed no effect on IAA, excluding the possibility of physical adsorption (Fig. 2g and h). Collectively, these results demonstrate that the O-HOF framework is essential for shielding the enzyme from degradation under harsh GI conditions. The integrated enzymatic and enzyme-mimicking functions for scavenging ROS and reducing metabolites establish MAO@NADH@PB@O-HOF@PEG as a comprehensive therapeutic nanoreactor, and the working principle is schematically summarized in Fig. 2i. The encapsulated MAO is responsible for the primary catalytic conversion of IAA. Concurrently, the PB component provides a ROS-scavenging cascade: its SOD-like activity catalyzes the dismutation of O_2_^·–^ to H_2_O_2_. The H_2_O_2_ is then eliminated by two catalytic pathways provided by the PB component: the CAT-like activity and the NPX-like activity, the latter utilizing the pre-loaded NADH to produce NAD^+^.

**Figure 3.**
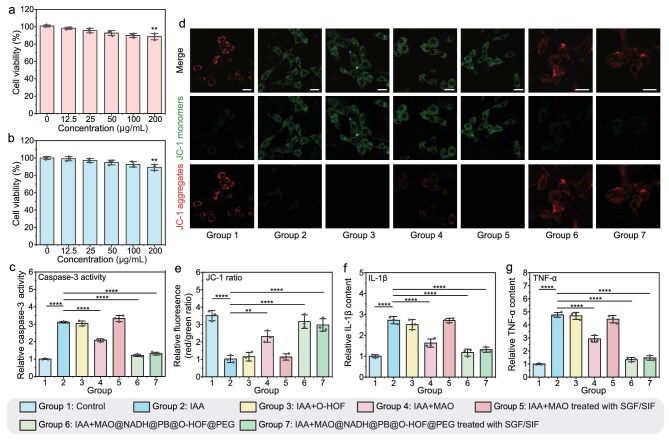
(a and b) Cell viability of (a) L929 cells and (b) CT26 cells treated with different concentrations of O-HOF. (c) Relative caspase-3 activity from the different treatment groups. (d) Images of mitochondrial membrane potential of cells stained with JC-1 after various treatments, and (e) the corresponding quantitative analysis. Scale bars = 25 μm. (f and g) Secretion levels of (f) IL-1β and (g) TNF-α in cell culture supernatants from the different treatment groups. Data represent mean ± SD (*n* = 3). (n.s., not significant, *P < 0.05, **P < 0.01, ***P < 0.001, ****P < 0.0001).

Safety is the prerequisite for long-term medication for chronic diseases. The evaluation of biocompatibility is the first criterion that must be determined before implementing biological applications. At the cellular level, we performed 3-(4,5-dimethylthiazol-2-yl)-2,5-diphenyltetrazolium bromide (MTT) assays to assess cytotoxicity. As shown in Fig. 3a and b, and Figs S14 and S15, O-HOF exhibited low cytotoxicity in L929, CT26 and HMC3 cells, with cell viability remaining above 85% at a concentration of 200 μg/mL after 24 h of incubation, and above 70% at 200 μg/mL after 48 h of incubation, demonstrating the favorable biocompatibility of O-HOF.

Building on the *in vitro* catalytic elimination of IAA (Fig. 2), we next investigated whether this biochemical activity could mitigate key pathological cellular mechanisms associated with IAA. It has been reported that IAA plays a role in inducing apoptosis in microglia [54]. Next, to explore whether IAA induces caspase-dependent apoptosis, caspase-3 activity in the treated HMC3 cells was determined by using a caspase-3 activity assay kit. As shown in Fig. 3c, the ‘IAA’ group showed a significant increase in caspase-3 activity compared to the control groups. The group treated with ‘IAA + MAO treated with SGF/SIF’ exhibited no discernible inhibition of caspase-3 activity, indicating that the catalytic function of MAO was deactivated under the harsh conditions in the absence of the protective O-HOF shell. While the ‘IAA + MAO’ group showed a partial decrease in caspase-3 activity, the effect was limited due to the compromised catalytic activity of the unprotected enzyme. Notably, caspase-3 activity remained substantially low after incubation with ‘IAA + MAO@NADH@PB@O-HOF@PEG treated with SGF/SIF’. Furthermore, treatment with ‘IAA + O-HOF’ did not show a discernible effect on caspase-3 activity, indicating that the observed therapeutic benefits are attributable to MAO catalytic activity rather than non-specific physical adsorption by the HOF scaffold. These findings demonstrate that the encapsulation of MAO within O-HOF effectively preserves the catalytic ability to suppress caspase-3 activity, underscoring the significant potential of MAO@NADH@PB@O-HOF@PEG for further applications.

Altered mitochondrial membrane potential is a marker of apoptosis and can be detected using the J-aggregate-forming lipophilic cation 1 (JC-1) probe [58]. In normal mitochondria, JC-1 accumulates in the mitochondrial matrix to form J-aggregates with strong red fluorescence. When the mitochondrial function in injured cells is disturbed, leading to a decrease in mitochondrial membrane potential, its membrane is dominated by the green fluorescent JC-1 monomer. Thus, the mitochondrial membrane potential was subsequently evaluated by utilizing the JC-1 probe. As shown in Fig. 3d and e, the control groups showed bright red fluorescence and high red-to-green ratio, which indicated a minimal change in the mitochondrial membrane potential. The ‘IAA’ group exhibited significantly reduced red-to-green fluorescence ratios, indicative of mitochondrial damage. Moreover, the unprotected MAO enzyme showed limited efficacy in restoring the mitochondrial membrane potential. The red-to-green fluorescence ratios were not restored by MAO treated with SGF/SIF, demonstrating that SGF/SIF-treated MAO was unable to ameliorate IAA-induced mitochondrial damage and apoptosis due to enzyme inactivation. Notably, incubation with SGF/SIF-treated MAO@NADH@PB@O-HOF@PEG led to a significant enhancement in red fluorescence intensity alongside a reduction in green fluorescence intensity, effectively restoring the red-to-green ratio. The ‘IAA + O-HOF’ control showed no detectable effect on mitochondrial potential, verifying that the observed recovery is driven by the enzymatic activity of the encapsulated MAO. These results suggest a potent therapeutic effect of the MAO@NADH@PB@O-HOF@PEG treatment, highlighting the critical role of the HOF framework in protecting its encapsulated cargo and retaining its catalytic efficiency.

As reported, in addition to promoting microglial cell apoptosis, IAA may also promote the accumulation of pro-inflammatory cytokines and contribute to neuroinflammation [54]. Following this, we then used enzyme-linked immunosorbent assay (ELISA) kits to measure the levels of these inflammatory cytokines. The results demonstrated that compared to the control group, the incubation with IAA significantly increased the release of inflammatory cytokines tumor necrosis factor-alpha (TNF-α) and interleukin-1 beta (IL-1β) in the supernatant (Fig. 3f and g). Notably, upon the treatment with MAO@NADH@PB@O-HOF@PEG, the levels of TNF-α and IL-1β were effectively reduced. The ‘IAA + O-HOF’ control had no impact on cytokine release, further supporting that the therapeutic effects derive specifically from nanoreactor-mediated catalysis. In contrast, the ‘IAA + MAO’ group exhibited only a partial reduction, while the ‘IAA + MAO treated with SGF/SIF’ group showed negligible therapeutic effect. This distinction underscores the protective effect of the O-HOF shell in maintaining enzymatic activity against harsh conditions. Beyond the metabolic regulation of IAA, we further validated the intracellular ROS-scavenging activity of the nanoreactor, which is attributed to the enzyme-mimetic properties of the PB component. Using a 2′,7′-dichlorodihydrofluorescein diacetate (DCFH-DA) assay, we found that MAO@NADH@PB@O-HOF@PEG significantly mitigated the increase in intracellular ROS in lipopolysaccharide (LPS)-stimulated HMC3 microglial cells (Fig. S16). This indicates the nanoreactor’s capacity to alleviate cellular oxidative stress.

Next, the prolonged intestinal retention of MAO@NADH@PB@O-HOF@PEG was evaluated by tracking and visualizing the fluorescence signal from cyanine 5 (Cy5)-labeled MAO@NADH@PB@O-HOF@PEG (denoted as Cy5@MAO@NADH@PB@O-HOF@PEG) in mice after administration. After an oral gavage of Cy5@MAO@NADH@PB@O-HOF@PEG for 3 h, the mice were sacrificed, and their intestines were collected to assess the retention of Cy5@MAO@NADH@PB@O-HOF@PEG in the GI tract (Fig. S17). Notably, the fluorescence retention in the intestine was prolonged compared to non-PEGylated Cy5@MAO@NADH@PB@O-HOF, demonstrating the potential of the PEG-decorated oral HOF nanoreactor to achieve a sustained therapeutic effect. We speculated that the enhanced accumulation could be attributed to the interaction of PEG with the intestinal mucus layer via interpenetrating network effects [43,44]. The above results suggested that MAO@NADH@PB@O-HOF@PEG could localize to the GI tract with prolonged retention, reflecting the stability of the O-HOF to protect cargo and the role of PEG in enhancing intestinal residence during oral delivery.

**Figure 4.**
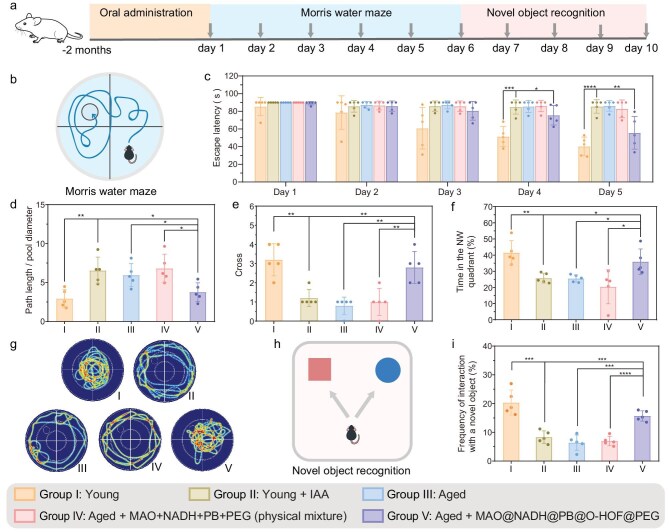
(a) Schematic illustration of the experimental timeline for oral administration and behavioral tests. (b) Schematic illustration of the MWM test. (c) Escape latency of mice during the 5-day MWM training phase. (d) The swim path length normalized to the pool diameter, (e) the number of platform crossings and (f) the percentage of time spent in the target (NW) quadrant. (g) Representative swim paths during the probe trial. (h) Schematic illustration of the NOR test. (i) Frequency of interaction with the novel object in the NOR test. Data are presented as mean ± SD (*n* = 5 mice per group). (n.s., not significant, *P < 0.05, **P < 0.01, ***P < 0.001, ****P < 0.0001). Part of this figure was adapted from SciDraw (DOI: 10.5281/zenodo.3925901).

We next investigated whether oral administration of the nanoreactor MAO@NADH@PB@O-HOF@PEG could alleviate cognitive dysfunction in aged mice. After 2 months of treatment administration via oral gavage (12.5 mg/kg for MAO@NADH@PB@O-HOF@PEG, every other day), the young and aged mice underwent behavioral tests (Fig. 4a). Morris water maze (MWM) experiments were performed to assess cognitive functions (Fig. 4b). This treatment was compared with a control group consisting of an unencapsulated physical mixture of MAO, NADH, PB and PEG (designated as Group IV in Fig. 4). During the training phase, untreated aged mice and IAA-treated young mice exhibited longer escape latencies compared to the young mice group, indicating impaired spatial learning (Fig. 4c). Notably, the escape latency of aged mice treated with MAO@NADH@PB@O-HOF@PEG (Group V) was reduced by Days 4 and 5, indicating an improvement of impaired cognitive functions. In the subsequent probe trial, the search path length of Group V-treated aged mice was shorter, indicating a more efficient memory-guided search (Fig. 4d). Furthermore, aged mice treated with Group V spent a greater percentage of time in the target (NW) quadrant (Fig. 4f) and exhibited more platform crossings (Fig. 4e) compared to the untreated and control-treated aged groups. The representative swim path showed a targeted search pattern for aged mice treated with MAO@NADH@PB@O-HOF@PEG (Fig. 4g). In contrast, aged mice in Group IV (physical mixture) failed to improve MWM performance (Fig. 4c–g), even though the administered doses were significantly higher than the equivalent components in the Group V nanoreactor. Next, to further assess recognition memory, we performed the novel object recognition (NOR) test (Fig. 4h). The results showed that aged mice treated with MAO@NADH@PB@O-HOF@PEG (Group V) exhibited a higher frequency of interaction with the novel object (Fig. 4i), indicating the restoration of recognition memory. Conversely, Group IV treatment failed to restore recognition memory (Fig. 4i). Collectively, these results from the MWM and NOR tests suggest that age-related cognitive impairment can be effectively ameliorated by oral Group V treatment and the protective function of HOF is essential for *in vivo* efficacy.

**Figure 5.**
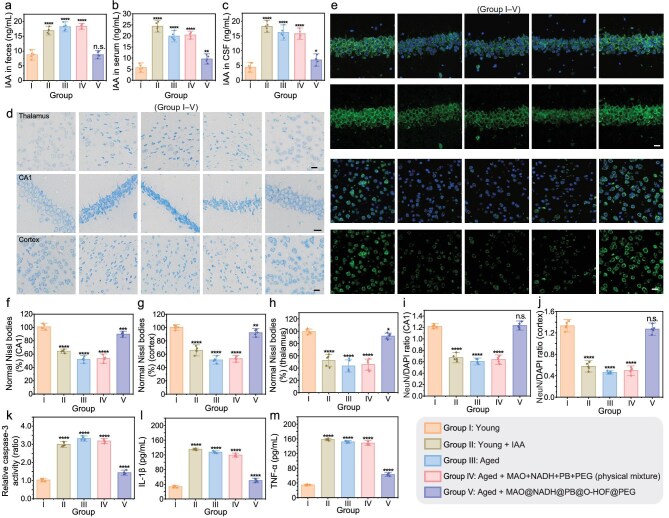
*In vivo* therapeutic efficacy of MAO@NADH@PB@O-HOF@PEG. (a–c) Quantification of IAA levels in the (a) feces, (b) serum and (c) CSF of mice. (d) Representative images of Nissl staining in the thalamus, hippocampal CA1 region and cortex. Scale bars = 20 μm. (e) Representative immunofluorescence images of neurons (NeuN, green) and nuclei (DAPI, blue) in the CA1 and cortex. Scale bars = 20 μm. (f–h) Quantification of the percentage of normal Nissl bodies in the (f) CA1, (g) cortex and (h) thalamus. (i and j) Quantification of the NeuN/DAPI ratio in the (i) CA1 and (j) cortex. (k–m) Measurement of (k) relative caspase-3 activity and the levels of inflammatory cytokines (l) IL-1β and (m) TNF-α in brain tissues. Data are presented as mean ± SD (*n* = 5 mice per group). (n.s., not significant, *P < 0.05, **P < 0.01, ***P < 0.001, ****P < 0.0001).

We next investigated the *in vivo* therapeutic efficacy of MAO@NADH@PB@O-HOF@PEG. First, we measured fecal IAA concentrations to assess their levels within the GI tract. The results revealed that aged mice and young mice orally administered IAA exhibited significantly elevated fecal IAA, compared to the young mice (Fig. 5a). Notably, oral administration of MAO@NADH@PB@O-HOF@PEG (Group V) significantly decreased the elevated IAA levels in aged mice, restoring the concentration to a level similar to that of the young control group. Furthermore, the elevated IAA levels observed in the serum and CSF of aged mice were also significantly reduced following treatment with Group V (Fig. 5b and c). In contrast, the free-mixture control (Group IV) failed to reduce IAA levels, which remained comparable to those of the untreated aged group. These findings indicate that MAO@NADH@PB@O-HOF@PEG treatment effectively reduced IAA levels in aged mice, demonstrating its potent *in vivo* catalytic activity.

Subsequently, histological analyses were conducted to investigate whether MAO@NADH@PB@O-HOF@PEG treatment could attenuate aging-related neuronal impairment. Nissl staining of brain sections indicated that aged mice, as well as young mice treated with IAA, exhibited significant neuronal loss and damage, as quantified by a reduced percentage of normal Nissl bodies (Fig. 5d and f–h). Notably, MAO@NADH@PB@O-HOF@PEG (Group V) treatment attenuated neuronal damage in the thalamus, hippocampal CA1 region and cortex of aged mice, significantly increasing the proportion of healthy neurons compared to the untreated aged group (Fig. 5d and f–h). This protective effect was not observed in Group IV, which exhibited significant neuronal loss similar to that of untreated aged mice. The therapeutic effect was further validated by hematoxylin and eosin (H&E) staining (Fig. S18). Next, we analyzed the expression of the neuronal marker neuronal nuclei (NeuN) in the hippocampal CA1 region and cortex. Significant neuron loss, evidenced by a markedly reduced density of NeuN-positive (green) cells, was observed in aged mice and in young mice treated with IAA. In contrast, aged mice treated with MAO@NADH@PB@O-HOF@PEG (Group V) exhibited a recovery of NeuN-positive neurons and elevated NeuN/DAPI ratio (Fig. 5e, i and j). Treatment with Group IV failed to induce such recovery. Collectively, these results demonstrate that MAO@NADH@PB@O-HOF@PEG treatment effectively mitigated the age-related increase in IAA and associated neuronal damage.

Previous studies have reported that elevated IAA levels contribute to microglial apoptosis and inflammation during aging [54,59,60]. Therefore, we investigated whether MAO@NADH@PB@O-HOF@PEG treatment could mitigate IAA-related apoptosis and inflammation in aged mice. As shown in Fig. 5k, caspase-3 activity was significantly elevated in aged mice and IAA-treated young mice, indicating increased apoptosis. In contrast, treatment with MAO@NADH@PB@O-HOF@PEG (Group V) substantially reduced caspase-3 activity in aged mice. Furthermore, the levels of inflammatory cytokines IL-1β and TNF-α were also elevated in both IAA-treated young and aged mice (Fig. 5l and m). Notably, MAO@NADH@PB@O-HOF@PEG treatment (Group V) effectively mitigated the production of these inflammatory cytokines. Aged mice treated with the free mixture (Group IV) did not exhibit a reduction in apoptosis or inflammation markers. Taken together, these results demonstrate that MAO@NADH@PB@O-HOF@PEG treatment alleviates age-associated apoptosis and neuroinflammation in the brain.

Since the systemic leakage of IAA is closely linked to intestinal permeability—which is often compromised by age-related oxidative stress [61,62], we further assessed the *in vivo* therapeutic effect of the nanoreactor’s ROS-scavenging activity on gut barrier function. Immunofluorescence staining for the tight-junction proteins zonula occludens-1 (ZO-1) and occludin revealed disrupted and discontinuous expression in aged mice (Fig. S19). Notably, oral treatment with MAO@NADH@PB@O-HOF@PEG effectively restored the continuous epithelial expression of these proteins. This finding demonstrates the *in vivo* efficacy of the nanoreactor in mitigating age-related gut barrier disruption driven by oxidative stress, which is crucial for reducing the leakage of gut-derived IAA into the systemic circulation.

Furthermore, H&E staining of major organs exhibited intact tissue structures with no notable difference compared with the control group (Fig. S20), thereby confirming the biocompatibility of MAO@NADH@PB@O-HOF@PEG.

## DISCUSSION

A central challenge in the oral administration of therapeutics, particularly biological compounds such as enzymes and peptides, lies in overcoming the formidable barriers of the GI tract, including extreme pH and proteolytic degradation [11,21,23]. Our work leverages the unique attributes of HOFs to address this challenge. Conventionally, oral delivery strategies have faced significant hurdles. Polymeric nanoparticles, while capable of encapsulation, often exhibit insufficient stability in the acidic milieu of the stomach, leading to premature ‘burst release’ and exposure of fragile biological compounds to intestinal proteases [63,64]. Lipid-based systems like liposomes are inherently unstable against bile salts and lipases, resulting in rapid degradation [65,66]. Similarly, enteric coatings, though clinically established, are primarily designed for drug release upon pH change rather than protecting enzymes for sustained *in situ* catalytic function [67–71].

To address these limitations, recent advances have introduced innovative and unconventional strategies. For instance, supramolecular assemblies, such as windchime-like cyclodextrins, have been developed to deliver antioxidant enzymes by leveraging amphiphilic self-assembly [72]. Advanced functional coatings have also shown promise; a chitosan-coated ‘intercoil’ (IC)-tagging platform was reported to significantly stabilize therapeutic peptides [73]. Furthermore, complex systems like dual-coated antioxidant Mn-MOFs have successfully delivered antibodies by combining pH-responsive polymers with mucosal adhesion [74]. In the realm of crystalline materials, a ‘cage encapsulation’ strategy utilizing metal-organic coordination cages has emerged to pre-encapsulate enzymes prior to crystallization, improving universality [75]. Furthermore, beyond synthetic carriers, engineered live biotherapeutics (probiotics) represent a cutting-edge approach to metabolize harmful gut metabolites, such as excess methionine, for disease intervention [76].

Despite these significant advances, the HOF-based nanoreactor presents distinct and complementary advantages over conventional and emerging platforms. In terms of stability and synthesis simplicity, the O-HOF scaffold demonstrates robust structural integrity, not only in simulated physiological fluids but also under extreme conditions such as concentrated acid (12 M HCl) and boiling water (Fig. 1c, d and f, Fig. S10). This intrinsic robustness surpasses that of most polymeric carriers [63–66,77,78] and distinguishes our system from recent coating-based strategies that rely on external protective layers [73,74]. The O-HOF protects delicate cargo without the need for additional enteric coatings or multi-step surface modifications. Moreover, compared to the sophisticated ‘cage encapsulation’ method, which requires stepwise synthesis [75], our HOF system is constructed via a mild, one-pot self-assembly process, offering a facile route for enzyme encapsulation [25,26,35].

Unlike delivery systems designed solely for cargo release—whether via traditional enteric coatings or responsive polymers [73,74]—our nanoreactor functions as a protective microenvironment that both shields the enzyme (MAO) and allows the substrate (IAA) to diffuse in and the product to diffuse out. Specifically, the long-range ordered mesopores (24.5 × 18.6 Å) favor substrate diffusion, effectively accelerating chemical transformations [48] (Fig. 2). This enables sustained, localized catalytic activity within the intestine, a functionality challenging for simple burst-release nanoparticles [63, 64,67–71].

Regarding clinical translation potential, the system presents a favorable safety profile and therapeutic paradigm. Compared to MOFs [36,74,79], HOFs are entirely composed of light non-metallic elements (C, H, O, N) assembled via hydrogen bonding and π–π stacking. This metal-free nature mitigates concerns regarding metal ion residue or accumulation toxicity associated with long-term oral administration [36,79], offering higher biosafety for clinical translation. Furthermore, recent breakthroughs have utilized engineered live biotherapeutics to metabolically reprogram the gut, such as degrading plasma methionine for homocystinuria treatment [76]. While highly effective, live therapeutics face challenges regarding colonization stability and potential biosafety risks. Our synthetic HOF nanoreactor mimics this ‘metabolic reprogramming’ strategy—specifically reducing aging-related IAA levels—but does so using a biocompatible, non-living material, thereby circumventing the risks of bacterial colonization.

Collectively, our findings establish HOFs as a promising platform for next-generation oral drug delivery. Consistent with previous studies associating IAA with cognitive dysfunction [54], our results demonstrate that the catalytic degradation of intestinal IAA levels alleviates age-related neuronal damage and neuroinflammation. The engineered nanoreactor represents an advance by transforming the gut from a hostile environment into a therapeutic site for sustained biocatalysis. This approach provides a new potent tool for modulating the gut metabolome and opens an exciting avenue for treating age-related and other systemic diseases through oral catalytic therapy.

## CONCLUSION

In summary, we designed and synthesized a HOF-based oral nanoreactor to intervene in senescence by metabolic reprogramming. The O-HOF scaffold exhibited exceptional stability in strong acid environments, simulated gastric and intestinal fluids, and under high-temperature conditions, demonstrating its potential as a robust candidate for oral delivery and gut-localized catalytic therapy. The protective framework enabled our oral nanoreactor, MAO@NADH@PB@O-HOF@PEG, to effectively decrease elevated IAA levels in the intestine, serum and CSF, and consequently mitigate the associated apoptosis and inflammation. Oral administration of MAO@NADH@PB@O-HOF@PEG to aged mice alleviated cognitive decline, neuronal damage and neuroinflammation. Our work will inspire the design and synthesis of functional HOFs as oral therapeutic agents.

## Supplementary Material

nwag074_Supplemental_File

## MATERIALS AND METHODS

Detailed experimental methods can be found in the online supplementary file.
