# Supplementary material for: An orally administrable hydrogen-bonded organic framework (HOF)-based nanoreactor to reprogram metabolism for senescence intervention
Source: Natl Sci Rev. 2026 Feb 3;13(7):nwag074. doi: 10.1093/nsr/nwag074 (PMC13105169; doi:10.1093/nsr/nwag074)
Supplement: nwag074_Supplemental_File [file nwag074_supplemental_file.pdf]

Supporting Information

An orally administrable hydrogen-bonded organic framework (HOF)-based nanoreactor to reprogram metabolism for senescence intervention

Haochen Zhang,[+1,2] Junlin Ya,[+1,2] Jinsong Ren,[1,2] and Xiaogang Qu[,1,2]*

1Laboratory of Chemical Biology and State Key Laboratory of Rare Earth Resource Utilization, Changchun Institute of Applied Chemistry, Chinese Academy of Sciences, Changchun, Jilin 130022, P. R. China.

2School of Applied Chemistry and Engineering, University of Science and Technology of China, Hefei, Anhui 230026, P. R. China.

**Email: xqu@ciac.ac.cn*

KEYWORDS: Hydrogen-bonded organic frameworks, senescence, oral catalytic therapy, gut metabolome

Reagents and Materials:

1,3,6,8-tetrakis (p-benzoic acid) pyrene (H4TBAPy) were purchased from Yanshen Technology Co., Ltd (Jilin, China). Nicotinamide adenine dinucleotide (NADH) were purchased from Aladdin (Shanghai, China). Dulbecco's modified Eagle's medium (DMEM), 1,4-dimethylpyridinium iodide, piperidine, and 3- [4,5-dimethylthiazol-2-yl]-2,5-diphenyltetrazolium-bromide (MTT) were purchased from Sigma-Aldrich (St. Louis, MO, USA). Mitochondrial membrane potential assay kit, NAD+/NADH assay kit was purchased from Beyotime Biotechnology (Shanghai, China). PVP were purchased from Shanghai Macklin Biochemical Co., Ltd. (Shanghai, China). 1-[3-(dimethylamino)propyl]-3-ethylcarbodiimide hydrochloride (EDC) and N-hydroxysulfosucnimide sodium (NHS) were purchased from Energy Chemical. Phosphate buffer solution (PBS) and Sulfo-Cy5 (SJ-MD0105) were purchased from Shandong Sparkjade Biotechnology Co., Ltd. Other chemicals were purchased from Macklin (Shanghai, China). Milli-Q water (18.2 MΩ; Millpore Co. USA) was used to prepare all buffers. Cell culture dishes and cell culture plates were bought from Guangzhou Jet Bio-Filtration Co., Ltd. All the chemical reagents were analytical grade and directly used without further purification unless otherwise specified.

Measurements:

Fourier transform infrared (FT-IR) analysis was carried out on a Bruker Vertex 70 FT-IR spectrometer. The scanning electron microscope (SEM) images were taken by a Hitachi S-4800 FESEM at working voltage of 10 kV and working current of 10 A. Ultraviolet-Visible (UV-Vis) absorption spectra were recorded with a JASCO-V550

spectrometer. Powder crystal X-ray diffraction (PXRD) was performed on Rigaku-Dmax 2500 diffractometer by using CuKα radiation. Adsorption isotherms of CO2 were measured using the ASAP 2020 from Micromeritics Co. Ltd. Zeta potential and dynamic light scattering (DLS) measurements were performed on a Zetasizer 3000HS analyzer. Fluorescence spectrum was obtained with a JASCO FP-6500 spectrofluorometer. An Olympus BX-51 optical equipped with a CCD camera was used for capturing fluorescence images. The high-performance liquid chromatography (HPLC) was measured by Ultimate 3000. The confocal laser scanning microscopy (CLSM) characterization was acquired by a (Nikon Eclipse Ni-E, Japan) top-of- the-line motorized upright. The flow cytometry data was obtained by BD LSRFortessa™ Cell Analyzer. Morris water maze (MWM) test was performed on WMT-100S Morris water maze (TECHMAN Co., LTD, Chengdu, China).

Methods

Synthesis of O-HOF: H4TBAPy (10 mg, 0.015 mmol) was dissolved in 1.5 mL of DMF to which 10 mL H_2O was added and stirred for 5 minutes. Then 8.3 mL of EtOH was added to the mixture. The products were separated via centrifugation at 12000 rpm for 15 minutes and further purified with EtOH and acetone for several times.

Preparation of Prussian blue nanoparticles (PB): $K_3[Fe(CN)_6]\cdot 3H_2O$ (131.72 mg) and PVP (3 g) were dissolved into HCl solution (0.01 M, 40 mL). After stirring for 30 min, the mixture was heated to 80 °C for 20 h. After aging for 24 h, the mixture was

centrifuged and washed with C_2H_5OH and double-distilled water three times to obtain pure PB.

Synthesis of MAO@NADH@PB@O-HOF: 20 mg H4TBAPy was dissolved into 2 mL DMF through ultrasonic treatment, followed by introducing 18 mL mixed aqueous solution containing MAO, NADH and PB under stirring at room temperature. After 5 min stirring, the mixed system was aged for another 10 min and allowed the formation. Finally, the precipitate was collected by centrifugation, washed by deionized water three times.

Synthesis of MAO@NADH@PB@O-HOF@PEG: The MAO@NADH@PB@O-HOF solution obtained above was adjusted to pH 6 by HCl (10%), then NHS and EDC were added into the solution and stirred at room temperature. The activated MAO@NADH@PB@O-HOF was then washed with DI water to remove unreacted NHS and EDC. The functionalized MAO@NADH@PB@O-HOF and PEG-NH_2 (2kDa) solution was sonicated for 15 min. Then, the above products were stirred together for 4 h. The ultimate product was washed by DI water.

Preparation of simulated gastric fluid and simulated intestinal fluid: The simulated gastric fluid (SGF) was obtained by dissolving 1% w/v porcine pepsin in prepared dilute hydrochloric acid solution with pH adjusted to 2.5, and simulated intestinal fluid (SIF) was obtained by dissolving 1% w/v porcine pancreatin in a solution containing 6.804 g L^{-1} KH_2PO_4 with the pH value of 6.8.

Quantification and Stability Analysis of MAO by SDS-PAGE: The loading concentration and stability of MAO were determined by SDS-PAGE with densitometric

quantification. To prepare samples, MAO@NADH@PB@O-HOF nanoreactors were boiled in 1× SDS-PAGE loading buffer (100 °C, 10 min) to release the encapsulated protein; this sample was used to determine the initial loading. For the stability assay, free MAO and MAO@NADH@PB@O-HOF (at equivalent initial MAO concentrations) were first sequentially incubated in SGF for 2h and SIF for 2 h. After incubation, these samples were also prepared by boiling in loading buffer. All samples were separated on a 12% SDS-PAGE gel alongside a standard curve of known MAO concentrations. Gels were stained with Coomassie Brilliant Blue. Band intensities were quantified using densitometry (ImageJ) and compared to the standard curve to determine the precise concentration of intact MAO in both the initial (untreated) and SGF-treated samples.

Guest inclusion study: Fluorescein (0.1 mM) or resorufin (0.1 mM) was mixed with O-HOF (0.5 mg mL^{-1}). The mixture was incubated at room temperature for 24 h before centrifugation at 14000 rpm for 20 min. The inclusion efficiency was calculated by measuring the fluorescent intensity of fluorescein (ex: 480 nm, em: 520 nm) or resorufin (ex:540 nm, em: 590 nm) in the supernatant.

Intracellular ROS Measurement: Intracellular ROS levels were measured using a 2′,7′-dichlorofluorescin diacetate (DCFH-DA) assay. HMC3 microglial cells were seeded in 96-well black-wall clear-bottom plates (or 12-well plates for imaging) and allowed to adhere overnight. The cells were then divided into three treatment groups: a Control group (treated with fresh medium), an Oxidative Stress group (stimulated with LPS at 1 μg/mL), and a Treatment group (co-treated with LPS at 1 μg/mL and

MAO@NADH@PB@O-HOF@PEG at 100 μg/mL). After incubation for 24 hours, the cells were washed with PBS and stained with DCFH-DA probe (10 μM) in serum-free medium in the dark for 30 min at 37°C. The intracellular green fluorescence, indicating ROS levels, was immediately captured using a fluorescence microscope and/or quantified using a microplate reader.

Measurement of the SOD-like activity: NBT, as the $\bullet O^{2-}$-sensitive probe, was used to evaluate the SOD-like activity. Different concentrations of MAO@NADH@PB@O-HOF@PEG were mixed with xanthine (0.6 mM) and xanthine oxidase (0.05 U/ml) in PBS buffer for 30 min. After centrifugation at 12000 rpm, NBT (100 μm) was added to the mixed solution to quantify the absorbance at 550 nm to evaluate the inhibition rate. For comparison, free PB nanoparticles, empty O-HOF, and samples sequentially treated with SGF/SIF were also evaluated under identical condition

Measurement of the CAT-like activity: The CAT-like activity of MAO@NADH@PB@O-HOF@PEG was evaluated by monitoring the generated oxygen from the decomposition of H_2O_2. Typically, MAO@NADH@PB@O-HOF@PEG (50 $\mu g \cdot mL^{-1}$) were mixed with 5 mM H_2O_2. The oxygen solubility was monitored by using a dissolved oxygen meter. For comparison, free PB nanoparticles, empty O-HOF, and samples sequentially treated with SGF/SIF were also evaluated under identical conditions. In addition to the dissolved oxygen measurement, the catalase-like (CAT-like) activity was further verified by monitoring the consumption of hydrogen peroxide (H_2O_2) using a titanium sulfate colorimetric method. Briefly, MAO@NADH@PB@O-HOF@PEG or control samples (including free PB and

samples treated with SGF/SIF) were mixed with a H_2O_2 solution to initiate the reaction. Subsequently, the reaction solution was centrifuged to remove the nanomaterials, and the supernatant was collected. A solution of Titanium(IV) sulfate was then added to the supernatant. The absorbance of the resulting solution was measured at 405 nm using a microplate reader.

Mimetic catalytic activity for the oxidation of NADH to NAD+: The mimetic catalytic activity in oxidizing NADH to NAD+ was evaluated using an NAD+/NADH assay kit (Beyotime, China) according to the manufacturer's instructions. Specifically, a reaction mixture containing MAO@NADH@PB@O-HOF@PEG (50 $\mu g \cdot mL^{-1}$ final concentration), NADH (0.2mM), H_2O_2 (10 mM final concentration) and HEPES buffer (50 mM pH 7.4) was prepared. After reaction, the generation of NAD+ and the consumption of NADH were quantified using a microplate reader according to the manufacturer's instructions. The NPX-like activity was expressed as the ratio of NAD+/NADH. For comparison, free PB nanoparticles, empty O-HOF, and samples sequentially treated with SGF/SIF were also evaluated under identical conditions.

HPLC analysis of microbial metabolites: The fecal samples were diluted with an equal volume of methanol. After centrifugation at 10,000x g for 30 min, 50 µl of supernatant was used for high performance liquid chromatography (HPLC) analysis.

IAA depletion: For the IAA depletion assay, MAO@NADH@PB@O-HOF@PEG and free MAO (normalized to equivalent protein concentration) were either left untreated or sequentially treated with SGF (2 h) and SIF (2 h). These samples, along with empty O-HOF (as a material control), were incubated with IAA solution in PBS

at room temperature. A mixture of IAA solution and PBS without any nanomaterials served as the control group. After incubation, the supernatant was collected, and IAA levels were determined by fluorometric assay with fluorescamine and HPLC.

Detection of Isoamylaldehyde Production: The generation of isoamylaldehyde was monitored using a colorimetric assay with 2-nitrophenylhydrazine. Briefly, the reaction supernatant was treated with 2-nitrophenylhydrazine solution to induce the formation of a hydrazone derivative. Following incubation to allow for color development, the absorbance of the resulting hydrazone derivative was measured at 372 nm using a microplate reader, serving as an indicator of isoamylaldehyde production.

Cell cultures: L929 cells and CT26 cells were supplied by ATCC (American Type Culture Collection). Cells were cultured in Dulbecco's modified Eagle's medium (DMEM) containing penicillin (100 U/mL), streptomycin (100 U/mL), and 10% fetal bovine serum (FBS) in a humidified incubator at 37 °C and 5% CO_2. Cells were harvested by the use of trypsin and were re-suspended in fresh complete medium before plating.

Cell viability studies: The viabilities of L929, CT26 and HMC3 cells were measured by MTT (3-(4,5-dimethyl-2-thiazolyl)-2,5-diphenyl-2-H-tetrazolium bromide) assay after being treated with different concentrations of O-HOF for 24 h or 48h.

Detection and Measurement of Inflammatory Cytokine Levels: HMC3 cells were seeded at a density of 1×10^5 cells per well and cultured overnight to allow for adherence. After the initial culture period, the medium was discarded, and each well was washed with PBS. The cells were then incubated with various formulations (including IAA,

MAO treated with or without SGF/SIF, MAO@NADH@PB@O-HOF@PEG treated with or without SGF/SIF and O-HOF) in DMEM for 12 hours. The supernatants were collected using centrifuge tubes. To quantify the levels of inflammatory cytokines TNF-α and IL-1β, enzyme-linked immunosorbent assay (ELISA) kits (Beijing Solarbio Science & Technology Co., Ltd.) were utilized.

Measurement of Mitochondrial Membrane Potential Following Treatment: HT22 cells were seeded at a density of 1.0×10^5 cells per well and cultured for 24 hours to allow for adherence. Subsequently, the cells were washed with PBS. The cells were then incubated with various formulations (including IAA, MAO treated with or without SGF/SIF, MAO@NADH@PB@O-HOF@PEG treated with or without SGF/SIF and O-HOF) in DMEM for 6 hours. After being washed with PBS, the cells were stained with JC-1 according to the manufacturer's protocol and imaged using a confocal laser scanning microscope (CLSM).

Animal Model: C57BL/6 (female) mice were purchased from the Laboratory Animal Center of Jilin University (Changchun, China).

Morris water maze (MWM) test:

Two-month-old (young) and 12-month-old (aged) male C57BL/6 mice (n=5) were administered via oral gavage of IAA (0.5 mg/kg of body weight) and physical mixture of MAO+NADH+PB+PEG (Group IV: 3 mg/kg MAO, 1 mg/kg NADH, 1 mg/kg PB, 6.5 mg/kg O-HOF, 1 mg/kg PEG) and MAO@NADH@PB@O-HOF@PEG (12.5 mg/kg) for different groups every other day for 2 months.

The Morris water maze was employed to assess memory function in these mice. Prior to the maze testing, animals underwent a training phase consisting of five trials per day for six consecutive days, with the platform position remaining fixed throughout. A tracking system automatically recorded the experimental data. The swimming pool was evenly divided into four sections, each trial began with the mouse positioned at one of four predetermined starting points. Mice could stay in pool for 90 seconds unless they found the platform. Once the mouse could not find the platform within the 90-second, the trials were terminated. The swimming speed, the percentage of time spent in the target quadrant relative to the total swimming time, the latency to find the platform, and the number of times the mouse passed over the platform position were recorded for statistical analysis.

Novel object recognition test: During the habituation phase, mice were allowed free exploration of a 40×40 cm open-field box for 5min. The arena was thoroughly cleaned with 70% (v/v) ethanol between mouse use. On the first training run, two identical objects were placed in opposite quadrants of the box and the mice were allowed to familiarize themselves with the objects for 10 min. After 24 h, one of the objects was replaced by a novel one of different color and shape but in the same location. Mice were removed from the cages and placed in the box. The time that mice spent in discovery of the familiar and the new object was recorded over a 10 min period. For four consecutive days, four trials (training) were recorded each day with a final test on day 5. The three- or four- day intersession interval is used to assess recognition memory performances.

***In Vivo* Histological Analysis:** For H&E staining, organs were initially fixed in 4% paraformaldehyde following saline treatment and then dehydrated. After paraffin embedding, sectioned, organs were subjected to H&E staining. For Nissl staining, the brains were separately collected. After preserving in 10% formalin, paraffin embedding, sectioned, and sectioned into 3 μm, these brain sections were then subjected to Nissl staining to detect Nissl bodies. For immunofluorescence staining, the entire brains were embedded in paraffin, then incubated with Triton X-100 for 10 min and blocked with BSA (0.5%) for 1 h at room temperature. Samples were then incubated with primary antibodies against NeuN (anti-NeuN antibody, green, 1:1000). After overnight incubation, the samples were incubated with Alexa Fluor®488-conjugated secondary antibodies for 1 h at 37 °C, counterstained with DAPI, then imaged using CLSM (Nikon Eclipse Ni-E) observation and quantitatively analyzed with ImageJ software. For immunofluorescence of intestinal tissues, intestinal tissues were collected, fixed in 4% paraformaldehyde (PFA), and embedded in OCT compound. The embedded tissues were cryosectioned at 10 μm thickness. The sections were permeabilized with 0.2% Triton X-100 in PBS for 10 min and then blocked with 5% BSA for 1 h at room temperature. The sections were subsequently incubated overnight at 4 °C with the following primary antibodies: anti-ZO-1 (1:200) and anti-Occludin (1:100). After washing with PBST, sections were incubated with the corresponding Alexa Fluor 488- and Alexa Fluor 594-conjugated secondary antibodies (1:500) for 1 h at room temperature in the dark. Nuclei were counterstained with DAPI. Images were acquired using a CLSM (Nikon Eclipse Ni-E).

Assessment of Inflammatory Levels in the Brain: To investigate the levels of inflammation factors TNF-α and IL-1β in the brains of mouse models after treatments, the brain of mice was collected and homogenized for 4 min in the 0.2 mL tube with RIPA buffer. Then the solution was centrifuged at 100,000 × g for 30 min. Lastly, the inflammatory mediators of the supernatants were tested by the corresponding enzyme-linked immunosorbent assay (ELISA) kits (Multi Sciences (Lianke) Biotechnology Co., Ltd (Beijing, China)).

Statistical Analysis: All outcomes were expressed as mean ± S.D. ($n = 3$ unless specified). Microsoft Excel, Origin 2020, and GraphPad Prism software were used to analyze data. We calculated the p-value through the student's t-test and labeled it in the graph. Asterisks indicated significant differences (*$P < 0.05$, **$P < 0.01$, ***$P < 0.001$, ****$p < 0.0001$)

Supporting Figures and Tables.

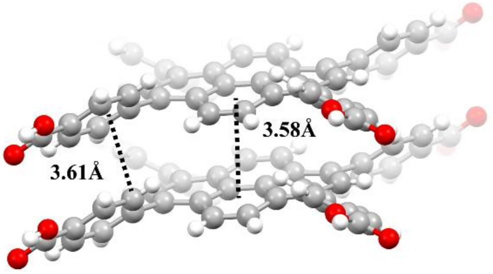

Figure S1. The face-to-face π-π interactions of crystal structure of O-HOF.

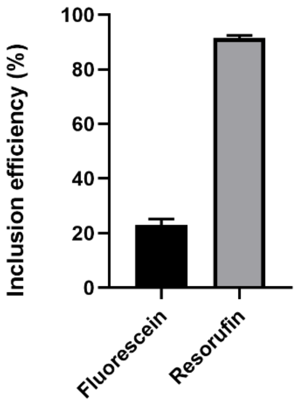

Figure S2. Guest inclusion assay of O-HOF.

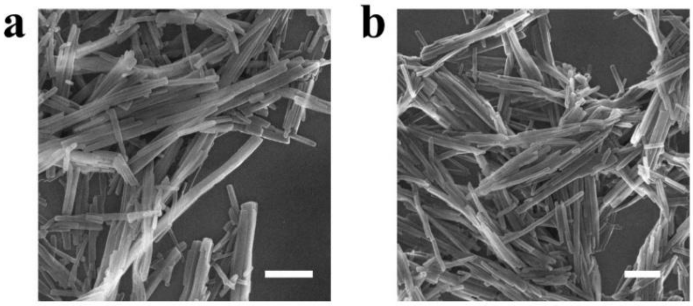

Figure S3. SEM images of O-HOF incubated in deionized water after 0 and 72 hours.

Scale bar = 600 nm.

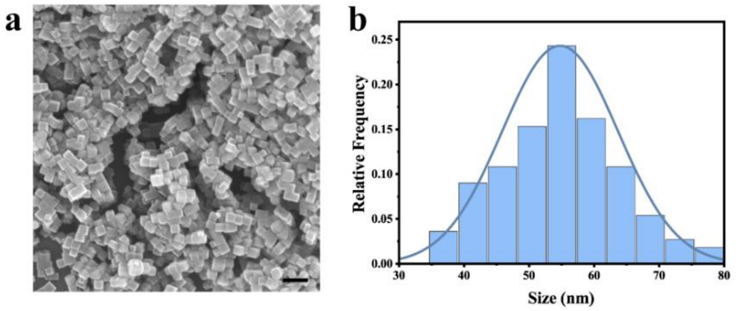

Figure S4. (a) SEM image of PB nanoparticles (scale bar: 200 nm) and (b) their corresponding size distribution.

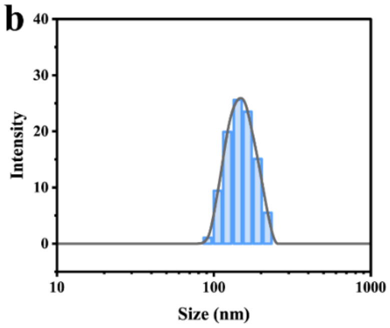

Figure S5. The DLS analysis of PB nanoparticles.

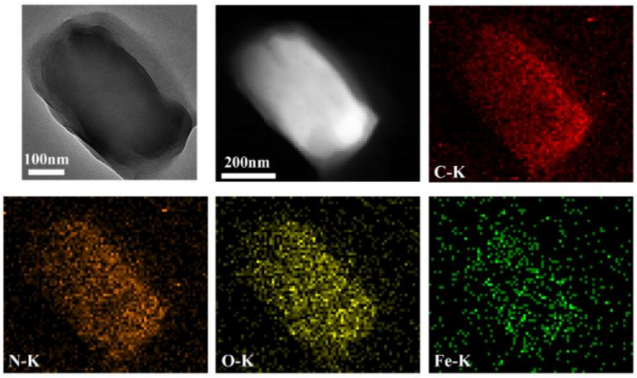

Figure S6. TEM image and corresponding EDS elemental mapping of MAO@NADH@PB@O-HOF, showing spatial co-localization of Fe (characteristic of PB) with the C, N, and O elements of the framework.

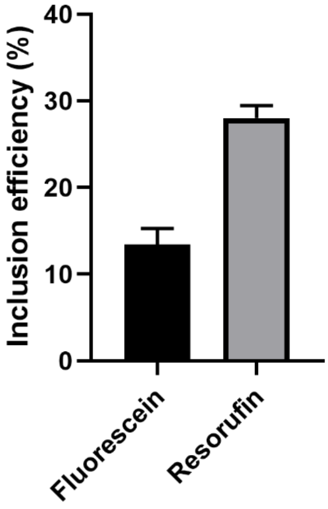

Figure S7. Guest inclusion assay of MAO@NADH@PB@O-HOF.

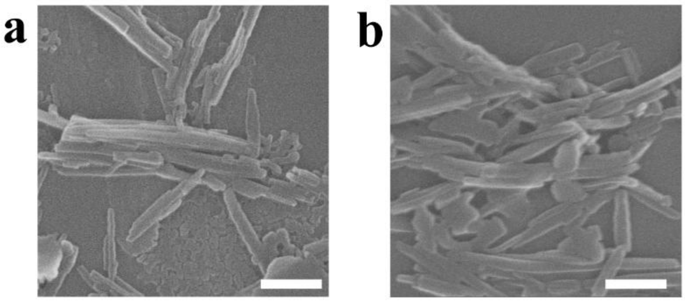

Figure S8. SEM images of MAO@NADH@PB@O-HOF incubated in deionized water after (a) 0 and (b) 72 hours. Scale bar = 300nm.

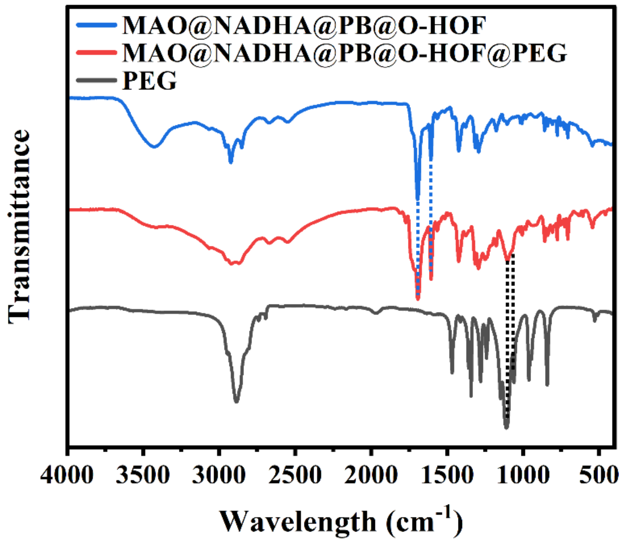

Figure S9. FTIR spectra of PEG, MAO@NADH@PB@O-HOF and MAO@NADH@PB@O-HOF@PEG.

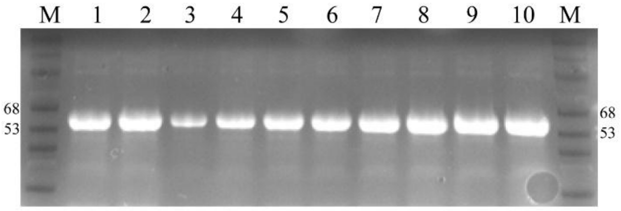

Figure S10. SDS-PAGE analysis of the encapsulated MAO. Lane 1 corresponds to MAO released from the nanoreactor MAO@NADH@PB@O-HOF@PEG after SGF/SIF treatment, while Lane 2 shows the MAO from the untreated nanoreactor. Lanes 3–10 represent a mass gradient (0.5 to 4.0 μg) of free MAO standards used for quantification.

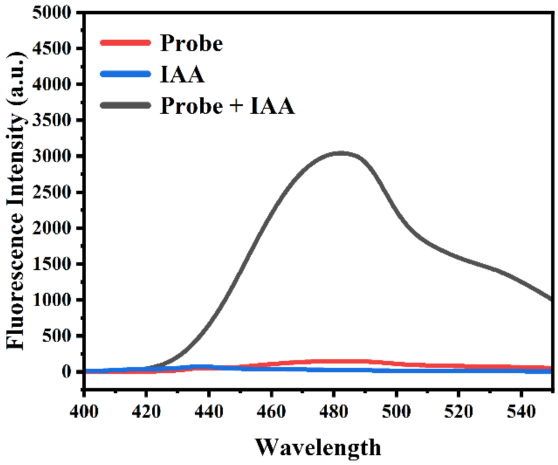

Figure S11. Fluorescence emission spectra of the probe (Fluorescamine) only, IAA only, and the probe incubated with IAA. A significant fluorescence signal was observed only in the presence of IAA due to the reaction between fluorescamine and primary amines. (Excitation wavelength: 380 nm).

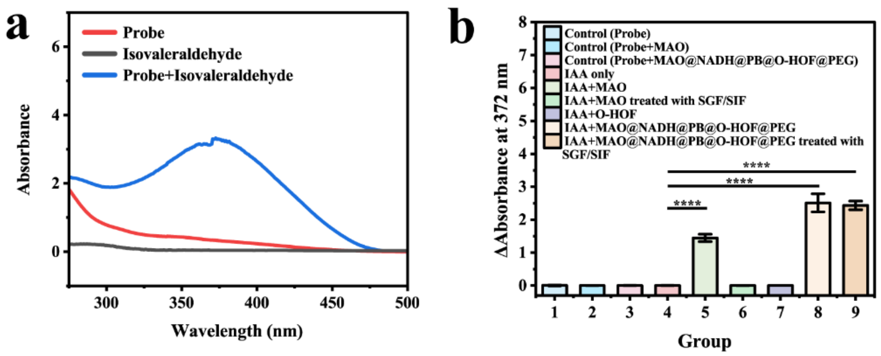

Figure S12. Generation and detection of isoamylaldehyde. (a) UV-vis absorption spectra verifying the reaction between the probe (2-nitrophenylhydrazine) and standard isoamylaldehyde. (b) Absorbance changes at 372 nm reflecting the catalytic conversion of isoamylamine (IAA) to isoamylaldehyde by free MAO and the MAO-loaded nanoreactor (MAO@NADH@PB@O-HOF@PEG). Data are presented as mean ± SD (n = 3). ****P < 0.0001.

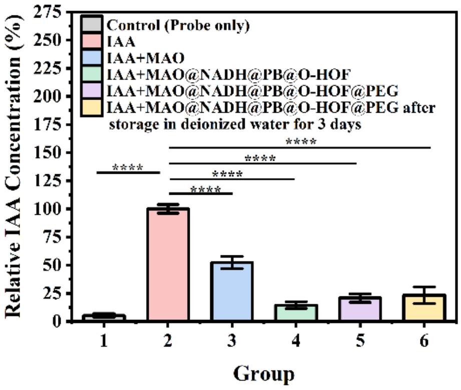

Figure S13. Catalytic activity and stability of the nanoreactors. IAA degradation efficiency was evaluated by monitoring fluorescence intensity changes for free MAO, encapsulated MAO, and PEGylated counterparts (at equivalent MAO concentrations determined by SDS-PAGE), either fresh or after 3-day storage. Data are presented as mean ± s.d. (n = 3, ****P < 0.0001).

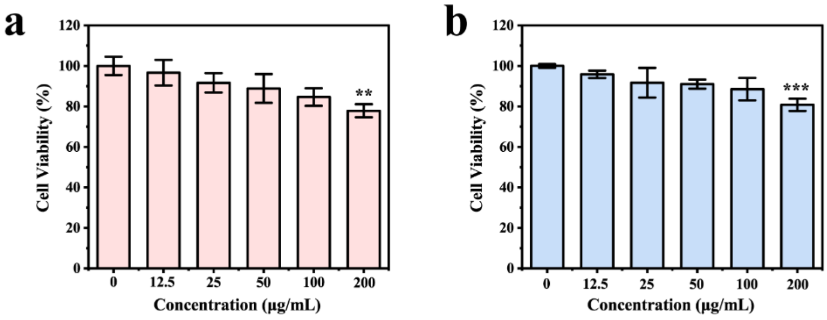

Figure S14. Viability of (a) L929 and (b) CT26 cells treated with O-HOF for 48h.

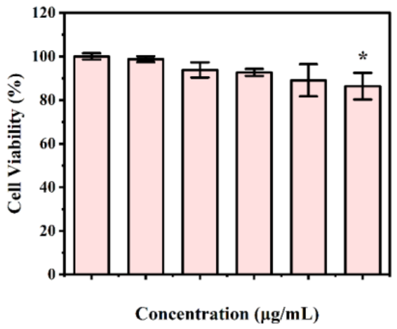

Figure S15. Cell viabilities of HMC3 cells treated with different concentrations of O-HOF.

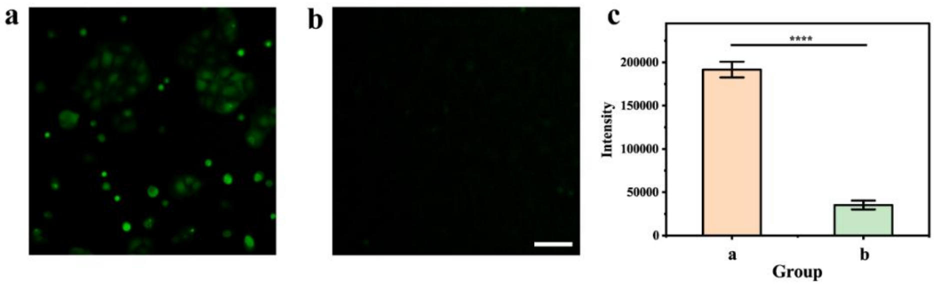

Figure S16. Intracellular ROS scavenging activity of MAO@NADH@PB@O-HOF@PEG in oxidative stress-induced HMC3 cells. (a) Fluorescence images of HMC3 cells stained with DCFH-DA, showing ROS under oxidative stress and (b) after treatment with MAO@NADH@PB@O-HOF@PEG. Green fluorescence indicates ROS. Scale bar = 100 μm. (c) Quantification of relative DCFH-DA fluorescence intensity. Data are mean ± s.d. (n = 3). (*P < 0.05, **P < 0.01, ***P < 0.001).

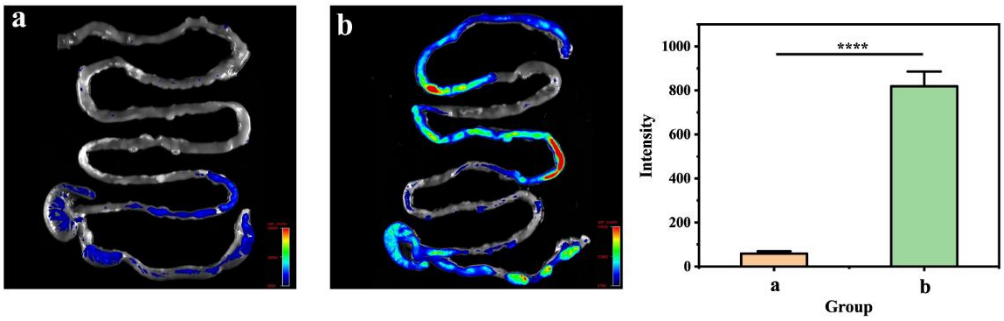

Figure S17. Fluorescent pictures of the whole GI tract 3 h post-oral gavage by (a) Cy5@MAO@NADH@PB@O-HOF or (b) Cy5@MAO@NADH@PB@O-HOF@PEG and corresponding quantitative analysis. (Data are presented as mean ± SD, n = 3, ****p < 0.0001)

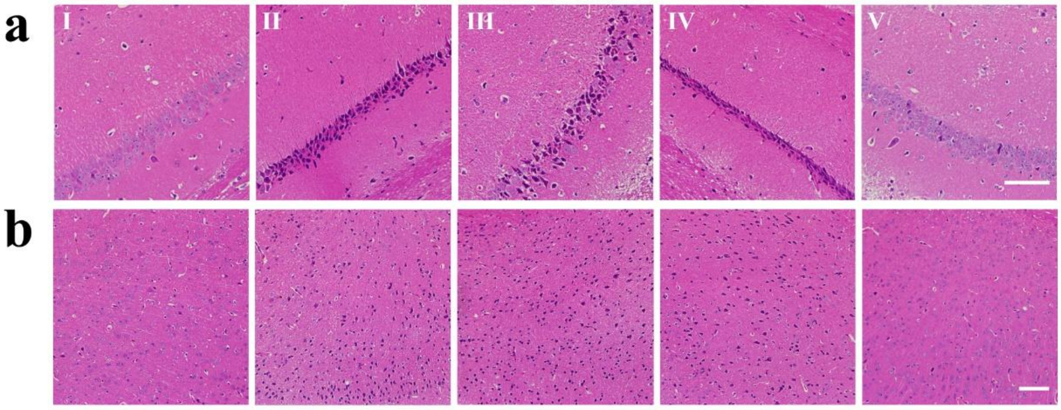

Figure S18. H&E staining of brain in (a) CA1 and (b) cortex in mice. Groups: (I) Young, (II) Young + IAA, (III) Aged, (IV) Aged + MAO+NADH+PB+PEG, (V) Aged + MAO@NADH@PB@O-HOF@PEG.

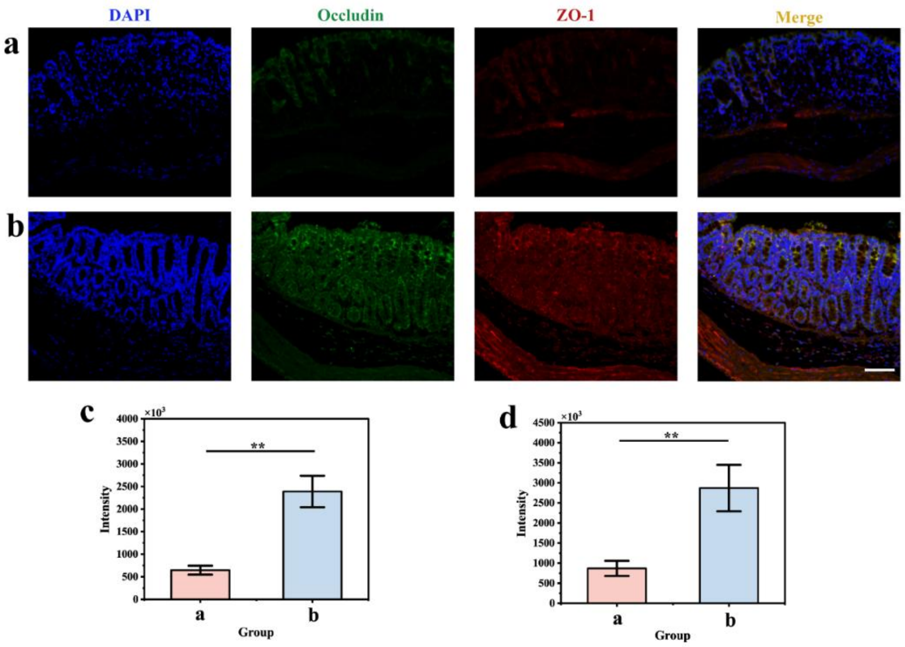

Figure S19. (a-b) Representative immunofluorescence images of tight-junction proteins (ZO-1, Red; Occludin, Green) in intestinal tissues from (a) aged mice and (b) aged mice treated with MAO@NADH@PB@O-HOF@PEG. Nuclei were counterstained with DAPI (Blue). Scale bar = 100 μm. (c-d) Quantitative analysis of (c) Occludin, (d) ZO-1. Data are mean ± s.d. (n = 3). (*P < 0.05, **P < 0.01, ***P < 0.001).

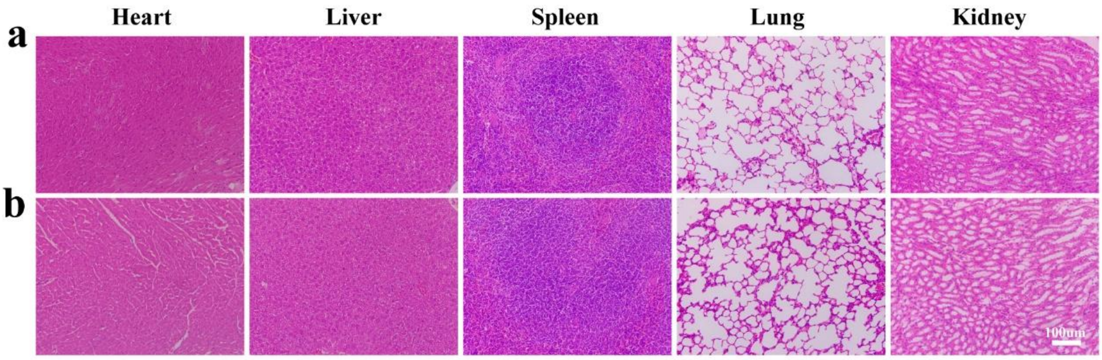

Figure S20. H&E staining of major organs in C57 mice treated with (a) PBS or (b) MAO@NADH@PB@O-HOF@PEG.
